# Increased Intrahepatic Expression of Immune Checkpoint Molecules in Autoimmune Liver Disease

**DOI:** 10.3390/cells10102671

**Published:** 2021-10-06

**Authors:** Zuzana Macek Jilkova, Marie Noelle Hilleret, Theophile Gerster, Nathalie Sturm, Marion Mercey-Ressejac, Jean-Pierre Zarski, Vincent Leroy, Patrice N. Marche, Charlotte Costentin, Thomas Decaens

**Affiliations:** 1 Université Grenoble Alpes, 38000 Grenoble, France; NSturm@chu-grenoble.fr (N.S.); mressejac@chu-grenoble.fr (M.M.-R.); jpzarski@chu-grenoble.fr (J.-P.Z.); patrice.marche@univ-grenoble-alpes.fr (P.N.M.); CCostentin@chu-grenoble.fr (C.C.); 2 Research Center Inserm U1209, Institute for Advanced Biosciences, CNRS UMR5309, 38700 La Tronche, France; 3 Service d’Hépato-Gastroentérologie, Pôle Digidune, CHU Grenoble Alpes, 38700 La Tronche, France; MNHilleret@chu-grenoble.fr (M.N.H.); tgerster@chu-grenoble.fr (T.G.); vincent.leroy2@aphp.fr (V.L.); 4 Reference Center for Inflammatory Biliary Diseases and Autoimmune Hepatitis (MIVB-H), French Network for Rare Liver Diseases in Children and Adults (FILFOIE), 75012 Paris, France; 5 Service d’Anatomo-Pathologie, Pôle de Biologie, CHU Grenoble Alpes, 38700 La Tronche, France

**Keywords:** autoimmune liver disease, autoimmune hepatitis, immune checkpoint molecules, PD-1, 4-1BB

## Abstract

Immune checkpoint molecules (ICM) are critical in maintaining immunologic homeostasis and participate in preventing or promoting autoimmune disease development. Exploring a large panel of intrahepatic inhibitory and stimulatory ICM is necessary for drawing a general picture of the immune alterations in autoimmune hepatitis (AIH). Here, we performed a multiparametric analysis of ICM, including PD-1, TIM3, LAG3, CTLA-4, OX40 and 4-1BB, and we determined their expression on intrahepatic lymphocyte subsets in untreated and in treated patients with AIH in comparison to normal liver tissue. AIH patient-derived liver tissue revealed the overexpression of ICM, mainly PD-1 and 4-1BB, as well as the strong correlation between PD-1^+^ CD8^+^ T-cell abundance and severity of AIH (alanine transaminase and aspartate transaminase levels). Our results show that the ICM play an important role in the loss of immune homeostasis in the liver, providing an attractive approach to investigate their role as targets for effective therapeutic interventions.

## 1. Introduction

Autoimmune liver diseases (AILD) are rare diseases characterised by immune-mediated hepatic injury. AILD have been mainly categorised into: (i) autoimmune hepatitis (AIH), (ii) primary sclerosing cholangitis (PSC) and (iii) primary biliary cholangitis (PBC). In AIH, the autoimmune injury targets mostly the hepatocytes, while in PSC the medium-sized intra- and extrahepatic bile ducts and in PBC interlobular bile ducts are affected [1]. Despite different patterns of inflammation, clinical phenotype, and outcomes, AIH, PSC and PBC are frequently grouped due to similarities in immunological markers, symptoms and treatment options. The incidence of AILD is increasing in certain countries [2,3,4]. Currently, the reported incidence rate of AIH ranges from 1 to 2.5 per 100,000 individuals in Europe. PBC and PSC are more common in northern than in southern Europe countries, and the reported incidence ranges from approximately 0.5 to 2.6 cases per 100,000 person-years [5,6,7,8].

AILD represent complex disorders characterized by an abnormal reactivity of the immune system against self-antigens. Compared to other forms of AILD, the immune alterations associated with AIH are relatively well described, but mostly at the peripheral blood level. AIH is characterized by loss of self-tolerance, which results from autoreactive CD4^+^ and CD8^+^ T cells and B cells and their abnormal activation and proliferation. T-cell mediated immune attack, which occurs during AIH, is caused by CD4+ T cells that secrete a range of pro-inflammatory cytokines, resulting in the recruitment and activation of other immune cells, and by cytotoxic CD8+ T cells that directly damage liver tissues [9,10]. Today, a majority of patients with AIH respond to standard immunosuppressive therapy with azathioprine and steroids. Still, insufficient response to standard therapy or serious side effects in 10–20% of AIH patients may require treatment changes [11]. The inflammatory process in AIH may be ameliorated and the immune tolerance could be potentially restored by targeting specific molecules involved in this abnormal autoimmune response.

Immune checkpoint molecules (ICM) are critical in maintaining immunologic homeostasis and participate in preventing or promoting autoimmune disease development. Mounting evidence demonstrates that impaired PD-1/PD-L1 function plays an important role in a variety of autoimmune diseases (as reviewed in [12,13]). In fact, PD-1, expressed primarily by T cells, directly inhibits T-cell proliferation and T-cell effector functions𠅌such as IFN-gamma production and cytotoxic activity - upon the ligation. Thus, this critical checkpoint counteracts immune stimulatory signals and limits cytotoxic T cells from tissue destruction. However, in autoimmune diseases, the PD-1/PD-L1 checkpoint pathway fails to stop autoimmune injury. Instead, PD-1^+^ cells infiltrate tissues, and this infiltration may escalate with disease progression, as has been shown in autoimmune encephalomyelitis [14].

In recent years, a growing number of new ICM have been discovered, and their implication in the pathogenesis of autoimmune diseases is currently investigated [15]. Exploring additional inhibitory and stimulatory ICM is necessary for drawing a general picture of the immune alterations in AIH. The intrahepatic expressions of different ICM in AIH were never reported. Here, we provide an exhaustive multiparametric analysis of immune-checkpoint expression on intrahepatic lymphocyte subsets in untreated and treated AIH in comparison to normal liver tissue showing a prominent link with liver pathology.

## 2. Materials and Methods

### 2.1. Patients and Liver Biopsy Processing

This study included 26 patients, who were enrolled between 2017–2021 either at diagnosis prior to any treatment initiation, or during clinical follow-up (Department of Gastroenterology and Hepatology, CHU Grenoble-Alpes). Detailed patient characteristics have been recorded as shown in Table 1. The AIH cohort consists of groups of AIH untreated (*n* = 11) and AIH during treatment (*n* = 5). AIH untreated patients had undergone liver biopsy as part of AIH diagnosis. AIH treated patients had undergone liver biopsy as part of medical follow-up to justify possible treatment withdrawal. The mean time between start of treatment and liver biopsy in group of AIH treated was 29.2 ± 8.6 months. The “Normal” control group includes liver biopsies from patients with suspected non-alcoholic fatty liver disease (NAFLD) where NAFLD or any other diagnosis was not confirmed (liver steatosis < 5%). Liver biopsies were divided into two parts; one part was used for histological examination, assessed by experienced liver pathologists, whereas the other part was processed within one hour following the clinical biopsy to conduct extensive phenotypic immunological analyses. The diagnosis of AIH was based on clinical (elevated alanine transaminase (ALT), aspartate transaminase (AST), etc.), immunological (positive smooth muscle antibody (SMA) and/or antinuclear antibody (ANA), etc.) and histological parameters (histological features compatible with AIH). All patients with PSC or PBC diagnosis were excluded. The study was performed in accordance with the Declaration of Helsinki and the French legislation based on local sample collection (DC-2014-2295), and all participants provided written informed consent.

### 2.2. Flow Cytometry Analyses

Immediately after the needle liver biopsy, freshly harvested liver tissue was transferred in the Hypothermosol™ FRS solution (4 °C), the weight of the tissue was determined and cells were recovered through mechanical disruption as previously [16,17]. Intrahepatic cell suspension was immunostained (without any stimulation), with the following anti-human antibodies of surface markers: Tube 1 included anti-CD45-APC/Cy7 (clone HI30, BioLegend, San Diego, CA, USA), anti-CD3-PerCP-Cy5.5 (clone UCHT1, BioLegend), anti-CD56-BV605 (clone HCD56, BioLegend), anti-CD16-AF700 (clone 3G8, BioLegend), anti-CD15-BV510 (clone W6D3, BioLegend), anti-CD8-PE/Cy7 (clone RPA-T8, BD Biosciences, Le Pont-De-Claix, France), anti-CD69-PE (clone FN50, BioLegend), anti-PD-L1-FITC (clone MIH1, BD Bioscience), anti-CTLA4-BV421 (clone BNI3, BioLegend) and anti-PD-1-BV711 (clone EH12.2H7, BioLegend). Tube 2 included anti-CD45-APC/Cy7 (clone HI30, BioLegend), anti-CD3-PerCP-Cy5.5 (clone UCHT1, BioLegend), anti-CD56-BV605 (clone HCD56, BioLegend), anti-CD16-AF700 (clone 3G8, BioLegend), anti-CD15-BV510 (clone W6D3, BioLegend), anti-CD8-PE/Cy7 (clone RPA-T8, BD Biosciences), anti-LAG3-PE (clone 3DS223H, eBioscience), anti-OX40-FITC (clone Ber-ACT35, BioLegend), anti-4-1BB-BV421 (clone 4B4-1, BioLegend) and anti-TIM3-BV785 (clone F39-2E2, BioLegend). The fluorescence minus one (FMO) controls, same as the corresponding isotype controls were used to determine positive populations. Zombie UV™ Fixable Viability kit was used to exclude dead cells. FluoroFix™ Buffer (Biolegend) was used for fixation to stabilize tandem dyes. Data were acquired on BD-LSRII flow cytometer (BD Biosciences), collected with BD FACSDiva 6.3.1 software and analyzed using FCS Express 6 Flow software.

### 2.3. Statistical Analysis

Analyses were performed using the statistical software GraphPad Prism 6 (GraphPad Software, San DiegoCity, CA, USA). Normal distribution was tested by means of the D’Agostino–Pearson omnibus normality test. When data were normally distributed, the unpaired t-test was used to determine significant differences observed between the groups. On the other hand, when data from either cohort were not normally distributed, the Mann-Whitney test was performed. The non-parametric Kruskal–Wallis one-way analysis of variance was used for multiple comparisons. *p* value < 0.05 was considered to be significant. Spearman correlation non-parametric test was conducted to determine the degree of correlation between variables when at least one variable’s data was non-normally distributed. *p*-values in correlation matrix were adjusted by Bonferroni correction method.

## 3. Results

### 3.1. The Number and Distribution of Intrahepatic Lymphocytes Are Modified in AIH

In this study, we analyzed 26 liver biopsies divided into following groups: (i) AIH untreated (*n* = 11), (ii) AIH treated (*n* = 5) and (iii) Normal (*n* = 10). The exact weight of the biopsy was determined (mean = 6.2 ± 0.14 mg of tissue) to begin with, followed by tissue homogenization. Fresh samples were stained using two different panels for the identification of the major lymphocyte populations and the expression of immune checkpoint molecules by flow cytometry.

**Figure 1:**
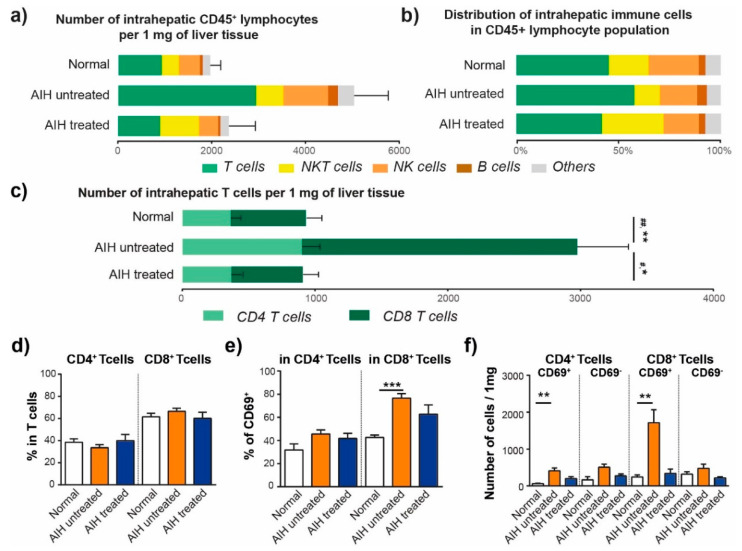
Number and distribution of intrahepatic lymphocytes is modified in AIH. (**a**) Estimated number of CD45^+^ lymphocytes per 1 mg of liver tissue. (**b**) Frequency of intrahepatic immune cells in CD45^+^ lymphocytes. (**c**) Estimated number of intrahepatic T cells per 1 mg of liver tissue. # *p* < 0.05, ## *p* < 0.01 represent statistically significant difference in number of CD4^+^ T cells between groups, * *p* < 0.05, ** *p* < 0.01 represent statistically significant difference in number of CD8^+^ T cells between groups. Kruskal-Wallis test with Dunn multiple comparison post-test. (**d**) Frequency of intrahepatic CD4^+^ T cells and CD8^+^ T cells in T cell population. (**e**) Frequency of intrahepatic CD69^+^ cells in CD4^+^ T cell and in CD8^+^ T cell population. (**f**) Estimated number of CD69^+^ or CD69^−^ CD4^+^ T cells and CD69^+^ or CD69^−^ CD8^+^ T cells per 1 mg of liver tissue. * *p* < 0.05, ** *p* < 0.01, *** *p* < 0.001between groups, Kruskal-Wallis test with Dunn multiple comparison post-test. Data are expressed as mean ± SEM. Normal (*n* = 10), AIH untreated (*n* = 11) and AIH treated (*n* = 5).

We investigated T, NK, NKT, and B cells adopting the strategy with the principle of gating as previously described [16,18,19]. The estimated number of cells per mg of tissue was calculated based on the original weight of the biopsy. As expected, the number of CD45^+^ lymphocytes per 1 mg was significantly higher in AIH untreated group (5025 ± 740, *p* = 0.0012) compared to Normal (1954 ± 241), which was mainly caused by a significant accumulation of T cells in AIH untreated group (2933 ± 494 of T cells per 1 mg, *p* = 0.0016) compared to Normal (924 ± 174), Figure 1a. Similarly, we observed an abnormal intrahepatic accumulation of B cells in the AIH untreated group (211 ± 52 of B cells per 1 mg, *p* = 0.0159) compared to Normal group (65 ± 9), Figure 1a. The intrahepatic accumulation of lymphocytes was normalized in the AIH treated group, showing 2369 ± 561 CD45^+^ lymphocytes per 1 mg of liver, 900 ± 175 T cells per 1 mg of liver and 50 ± 15 B cells per 1 mg of liver. Concerning the estimated number of NK and NKT cells per mg of tissue, we observed no statistically significant differences between the groups.

The overall distribution of intrahepatic immune cells in CD45^+^ lymphocyte population differed between groups in this study. CD3^+^CD56^−^ T cells found in the AIH untreated biopsies accounted for more than 57.7 ± 3.6% of all CD45^+^ lymphocytes, whereas in Normal group, T cells represented only 45.2 ± 4.4% (*p* = 0.0390). On the other hand, CD3^+^ CD56^+^ NKT cells and CD3^−^CD56^+^ NK cells represented a non-significantly smaller population in AIH untreated group (12.5 and 18.2%) compared to Normal liver (19.5 and 24.4%), Figure 1b, even though their absolute numbers per tissue were similar (Figure 1a). The analysis of tissue from AIH patients during treatment revealed that both the number and distribution of intrahepatic immune cells were normalized (Figure 1a,b).

### 3.2. Activated CD8^+^ T Cells Are Accumulated in the Liver of Patients with AIH

The estimated number of CD4^+^ T cells per 1 mg of tissue was significantly increased in AIH untreated group compared to Normal group (893 ± 133 vs. 365 ± 76 of CD4^+^ T cells per 1 mg, *p* = 0.0033, Figure 1c). Similarly, we observed a dramatic accumulation of CD8^+^ T cells per 1 mg of tissue in AIH untreated group compared to Normal group (2040 ± 382 vs. 559 ± 101 of CD8^+^ T cells per 1 mg, *p* = 0.0020, Figure 1c).

Importantly, the number of intrahepatic CD4^+^ T cells and CD8^+^ T cells was normalized in AIH patients during treatment, with significant decrease in number of cells when compared to AIH untreated group, Figure 1c. The ratio of intrahepatic CD4^+^ T cells to CD8^+^ T cells was similar in all groups, with CD8^+^ T cells representing approximately 65% of the T cell population (Figure 1d).

The massive increase of number of CD8^+^ T cells (Figure 1c) was associated with significantly higher frequency of CD8^+^ T expressing activation marker CD69 in the AIH untreated group compared to Normal group (76.6 ± 3.9 vs. 42.6 ± 2.1% of CD69^+^ cells per CD8^+^ T cells, *p* = 0.0020, Figure 1e). Based on the estimated number of cells, untreated AIH is characterized by the accumulation of both, activated CD69^+^ CD4^+^ and CD69^+^ CD8^+^ T cells in the liver, while the population of CD69^−^ T cells does not statistically differ between groups (Figure 1f).

### 3.3. High Frequency of PD-1^+^ and 4-1BB^+^ T Cells in the Liver of Patients with AIH

**Figure 2:**
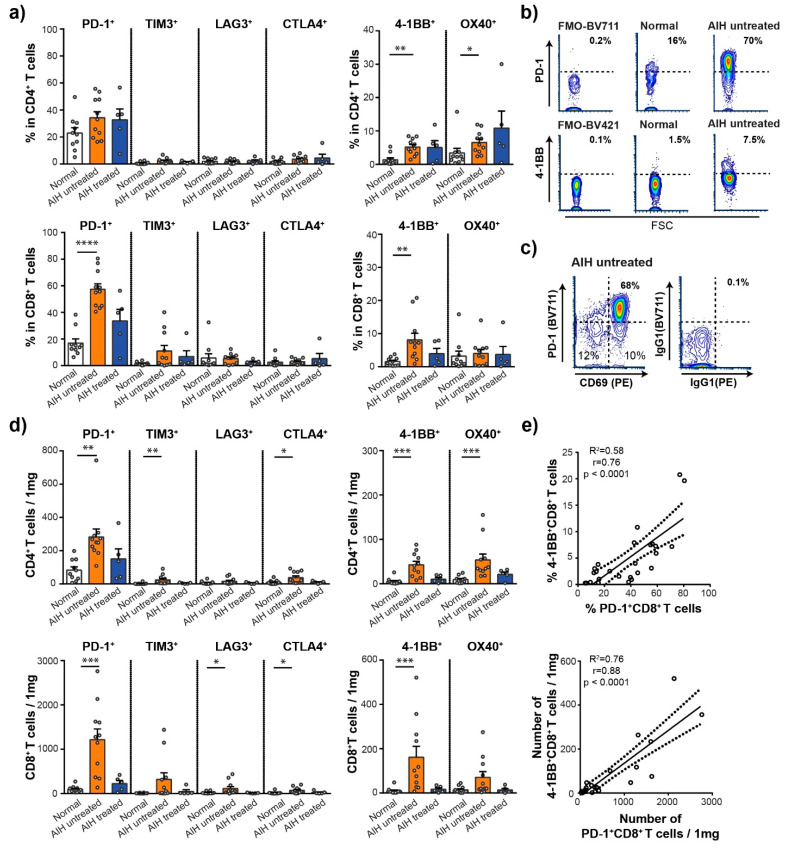
Immune checkpoint molecule expression on intra-hepatic CD8 and CD4 T cells in different subgroups. (**a**) The frequency of intrahepatic immune checkpoint molecule positive CD4^+^ and CD8^+^ T cells. Normal (*n* = 10), AIH untreated (*n* = 11) and AIH treated (*n* = 5). Each circle represents a patient. Data are expressed as mean ± SEM, * *p* < 0.05, ** *p* < 0.01, **** *p* < 0.0001 between groups (Kruskal-Wallis test with Dunn multiple comparison post-test). (**b**) Representative flow cytometry contour plot showing the expression of intrahepatic PD-1 on CD8^+^ T cells and 4-1BB on CD4^+^ T cells in fluorescence minus one control sample (FMO), in normal liver biopsy and in biopsy from patient with untreated AIH. (**c**) Representative flow cytometry contour plot showing the co-expression of PD-1 and CD69 in intrahepatic CD8^+^ T cells of patient with untreated AIH. (**d**) An estimated number of intrahepatic immune checkpoint positive CD4^+^ T cells and CD8^+^ T cells per 1 mg of liver tissue. Normal (*n* = 10), AIH untreated (*n* = 11) and AIH treated (*n* = 5). Each circle represents a patient. Data are expressed as mean ± SEM, * *p* < 0.05, *** *p* < 0.001 between groups (Kruskal-Wallis test with Dunn multiple comparison post-test). (**e**) The correlation between the frequency of 4-1BB^+^ and PD-1^+^ CD8^+^ T cells (upper graph) and the correlation between the number of 4-1BB^+^ and PD-1^+^ CD8^+^ T cells (lower graph). (R^2^) R-squared, (r) Spearman correlation coefficient. Each circle represents a patient.

Next, we analyzed the distribution of immune checkpoint molecules on the cell surface of CD4^+^ and CD8^+^ T cells, including inhibitory PD-1, TIM3, LAG3, CTLA4 and stimulatory 4-1BB and OX40 molecules (Figure 2).

The frequency of CD4^+^ T cells expressing stimulatory checkpoint molecule 4-1BB and OX40 was significantly higher in AIH untreated group compared to Normal group (4-1BB^+^ CD4^+^ T cells: 5.2 ± 0.7% vs. 1.4 ± 0.6%, *p* = 0.0010, OX40^+^ CD4^+^ T cells: 6.6 ± 0.9% vs. 3.4 ± 1.4%, *p* = 0.0443), while the inhibitory immune checkpoint molecules were similarly expressed by CD4^+^ T cells from different groups. Intrahepatic CD8^+^ T cells were characterized by a significantly higher frequency of cells expressing PD-1 in AIH untreated group, compared to Normal (57.4 ± 4.2% vs. 17.0 ± 3.1%, *p* < 0.0001), the same as a higher frequency of CD8^+^ T cells expressing 4-1BB^+^ (8.1 ± 2.0% vs. 1.5 ± 0.4%, *p* = 0.0011, Figure 2a,b). Importantly, PD-1 was almost exclusively co-expressed with CD69 activation marker on CD8^+^ T cells in AIH patients (Figure 2c).

The analysis, focused on estimated numbers of T cells expressing ICM per 1 mg of tissue, revealed that PD-1, TIM3, CTLA4, 4-1BB and OX40 positive CD4^+^ T cells are highly accumulated in AIH untreated liver compared to normal liver tissue. Similarly, we observed higher numbers of PD-1, LAG3, CTLA4 and 4-1BB positive CD8^+^ T cells in untreated AIH patients compared to Normal group (Figure 2d). Since we analyzed the markers of immune checkpoint receptors in two separated tubes, we cannot provide data pertaining to their co-expression per individual cell. However, we specifically investigated the co-expression of 4-1BB with other markers in 4 AIH patients, and our data revealed that 4-1BB is expressed on cells positive for PD-1 and CD69 (Supplementary Figure S1a). Moreover, the positive correlation between both the frequency and the number of CD8^+^ T cells expressing 4-1BB or PD-1 supports the co-expression of those receptors, as shown in Figure 2e.

Analyses targeting intrahepatic NK, NKT and B cells did not show any significant differences in ICM expression between groups. In addition, the distribution of intrahepatic NK cells into subpopulations of CD16^−^ and CD16^+^ was similar between all groups (Supplementary Figure S1b–d).

### 3.4. Intrahepatic T Cells Correlate Positively with Markers of Liver Pathology

**Figure 3:**
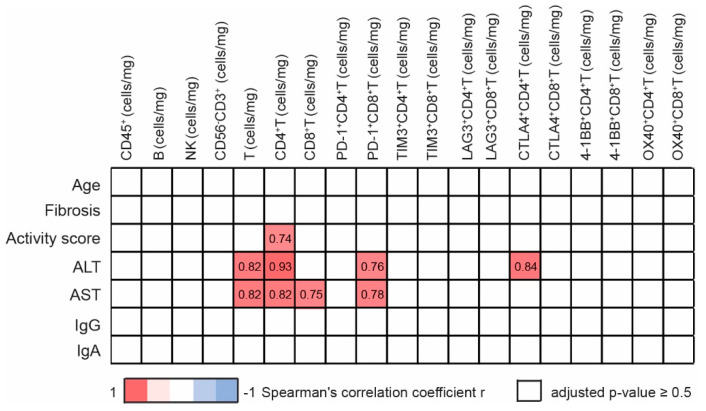
Correlation of patient characteristics and intrahepatic lymphocyte characteristics in untreated AIH group. Only significant correlations (Bonferroni-corrected *p* value < 0.5) are reported, numbers correspond to Spearman correlation coefficient r, positive correlation (red), negative correlation (blue), *n* = 11.

Next, we investigated the possible association between clinical data and lymphocyte characteristics in the group of untreated AIH patients. First, correlation analysis revealed a positive association of ALT and AST levels with the number of intrahepatic T cells (Figure 3).

A positive association of activity score, ALT and AST levels was also observed with the number of intrahepatic CD4^+^ T cells. Similarly, AST levels positively correlated with intrahepatic CD8^+^ T cells, Figure 3. Strong positive correlation was also observed between the number of intrahepatic PD-1 ^+^ CD8^+^ T cells and ALT and AST levels. The number of intrahepatic CTLA-4^+^ CD4^+^ T cells correlated positively with ALT levels.

Surprisingly, correlation analysis revealed no association of activity score or AST and ALT levels with number of intrahepatic PD-1^+^ CD4^+^ T cells, suggesting the different role of intrahepatic PD-1^+^ CD4^+^ T cells and PD-1^+^ CD8^+^ T cells in the immune-pathogenesis of AIH. Similarly, no association was observed between clinical data and number of 4-1BB^+^ T cells in the group of untreated AIH patients.

The importance of intrahepatic PD-1^+^ CD8^+^ T cells was confirmed when the association between clinical data and lymphocyte characteristics was analyzed in whole cohort (Supplementary Figure S1e).

## 4. Discussion

**Figure 4:**
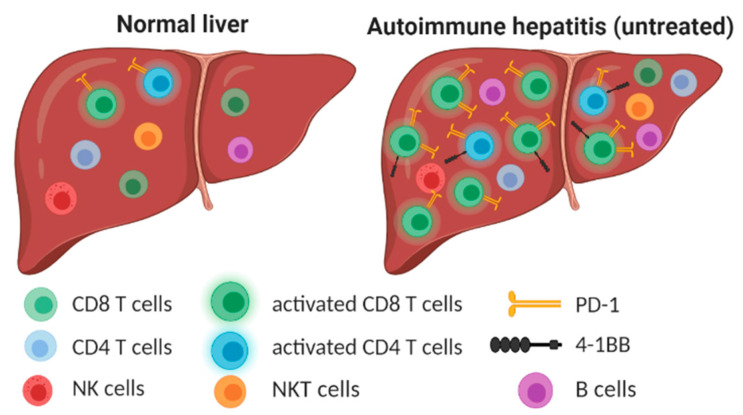
Untreated AIH is characterized by high accumulation of activated T cells with increased expression of PD-1 and 4-1BB immune checkpoint molecules. Created with BioRender.com.

In the present study, we performed multiparametric flow cytometry analysis based on fresh liver biopsy samples from patients with AIH. We analyzed a large panel of intra-hepatic ICM (PD-1, TIM3, LAG3, CTLA-4, OX40, 4-1BB) expressed by different immune cells, and demonstrated that untreated AIH patients are characterized by a high frequency of activated intrahepatic T cells expressing PD-1 and 4-1BB, Figure 4. The analysis of tissue from AIH patients during treatment revealed that both the number and distribution of intrahepatic immune cells are normalized during treatment while the expression of ICM seems to be normalized only partially.

Importantly, the accumulation of PD-1^+^ CD8^+^ T cells in the untreated AIH patient-derived liver tissue correlated positively with liver pathology (ALT and AST levels). The possible involvement of PD-1/PD-L1 pathway in the pathogenesis of AIH was previously described in mice model of experimental AIH [20] and in human studies [21,22]. Our results are in accordance with Oikawa et al., who previously demonstrated by immunohistochemistry that PD-1 ICM is expressed on more than half of the intrahepatic T cells in patients with AILD [21]. Similarly, a recent study showed a high frequency of PD-1 positive cells within liver tissue in children with AIH [22]. However, neither of these studies determined the exact type of intrahepatic T cells that express the PD-1 molecule. Circulating levels of PD-1 are also increased in AILD, as recent studies have shown that circulating levels of soluble PD-1 are elevated in AIH patients [23,24]. In addition, it was demonstrated that PD-1 and CD38 co-expression by circulating memory CD45RA^−^ CXCR5^−^ CD127^−^ CD27^+^ T cells reflects AIH activity [25].

Compared to previous studies, we analyzed intrahepatic lymphocytes by multiparametric flow cytometry, which allowed us to differentiate individual sub-populations of immune cells expressing PD-1 in liver. Here, we reveal the importance of CD8^+^ T cells and the positive correlation between the intrahepatic accumulation of PD-1^+^ CD8^+^ T cells and AIH severity. On the other hand, we observed that the number of PD-1^+^ CD4^+^ T cells was not associated with AIH severity, suggesting the different role of intrahepatic PD-1^+^ CD4^+^ T cells and PD-1^+^ CD8^+^ T cells in the immune-pathogenesis of AIH. Indeed, it was reported that PD-1 signaling promotes immunosuppressive functions of CD4^+^ T cells, mainly of regulatory Foxp3^+^ CD4^+^ T cells [26]. Small liver biopsy size did not allow us to analyze the intracellular expression of Foxp3^+^, which is a limitation of this study. Indeed, Foxp3^+^ CD4^+^ T cells are known to be enriched in the liver of untreated AIH patients [27,28] and decrease during therapy [28], showing an important role of this subpopulation of CD4^+^ T cells in liver. In future, it will be important to reveal the role of intrahepatic PD-1^+^ CD4^+^ Treg cells, their features, same as their proliferation and apoptosis status in autoimmune liver injury.

A growing body of evidence highlights the key role of the T cell costimulatory receptor 4-1BB for immune homeostasis. 4-1BB-deficient mice have impaired T-cell survival, proliferation and cytotoxicity [29]. 4-1BB-mediated T cell stimulation as measured by enhanced T cell proliferation and cytokine production can be induced by anti-4-1BB monoclonal antibodies or by employing the 4-1BB ligand. Today, agonist antibodies targeting 4-1BB are among the most effective immunotherapeutic agents across preclinical cancer models. However, early clinical studies have revealed that 4-1BB agonists can trigger high-grade liver inflammation. Thus, the development of these agents has been hampered due to the risk of severe liver toxicity [30]. Despite the strong capacity of 4-1BB activation to trigger hepatotoxicity, only a few studies have focused on the possible role of 4-1BB in AILD. A previous study showed a high level of intrahepatic 4-1BB in a mouse model of concanavalin (Con) A-induced immune-mediated liver injury. Importantly, blocking anti-4-1BB monoclonal antibodies attenuated the liver pathology in this animal model [31]. Bartkowiak et al. demonstrated recently that 4-1BB agonist antibodies trigger hepatitis via activation of 4-1BB on liver myeloid cells, which leads to CD8 T cells infiltration, tissue damage and transaminase elevation [32]. Thus, the expression of 4-1BB on liver myeloid cells in AILD deserves to be further investigated. Altogether, these results suggest that the inhibition of the 4-1BB pathway might be the next step toward new therapeutic approaches to control AIH. Paradoxically, the stimulation of 4-1BB with agonist antibodies also inhibits inflammation in many murine models of autoimmunity, which may be due to augmentation of regulatory CD8 T cell activity and/or driving the death of pathogenic CD4 T cells. Our study revealed that the frequency of intrahepatic 4-1BB positive T cells expression is high in AIH without any correlation with severity of AIH. Further investigation based on a larger number of patients is necessary to investigate possible association between clinical data and 4-1BB.

There are only a few studies addressing the involvement of other ICM in the pathology of AIH. For instance, the possible link of defective TIM3 pathway in AIH was identified previously on circulating lymphocytes [33]. Here, we have shown the significant accumulation of intrahepatic T cells expressing not only PD-1 and 4-1BB, but also TIM3, LAG3, CTLA4 and OX40 in AIH when compared to normal liver, and a possible association between their expression and liver pathology.

In summary, analysis of AIH patient-derived liver tissue revealed the expression of ICM and the link with liver pathology, as well as the strong correlation between PD-1^+^ CD8^+^ T-cell abundance and AIH severity. Recently, Zhao et al. showed that the depletion of PD-1^+^ cells may ameliorate autoimmune disease in different mouse models [34], but the manageable selectivity of treatment for CD8^+^ vs. CD4^+^ T cells should be carefully investigated.

Our results clearly show that the ICM are highly expressed in AIH and play an important role in the loss of immune homeostasis in the liver, providing an attractive approach to investigate their potential role as targets for effective therapeutic interventions.

## Figures and Tables

**Table 1:** Clinical, biological and histological features of patients.

	Normal	AIH Untreated	AIH Treated
Number of individuals	10	11	5
Sex, male/female	6/4	5/6	2/3
Age, y (mean ± SD)	47.3 ± 4.8	48.5 ± 7.3	51.6 ± 6.8
ALT, U/L (median; range)	33; 17–79	327; 44–2279	47; 25–836
AST, U/L (median; range)	29; 20–71	337; 31–982	55; 21–1212
IgG, g/L (median; range)	-	15.4; 12.3–22.2	12.8; 10.9–34.2
IgA, g/L (median; range)	-	3.1; 1.6–4.5	3.0; 2.8–4.6
SMA^+^ titer (>1:80), Yes/No (median; range)	-	9/3 (160; <40–1280)	2/3 (80; <40–640)
ANA^+^ titer (>1:80), Yes/No (median; range)	-	10/1 (320; <80–1280)	4/1 (160; <40–320)
Treatment			
None	10	11	-
Steroids/Steroids + Azathioprine	-	-	2/3
Histology, METAVIR scoring			
Fibrosis stage (F0/F1-F2/F3-F4)	9/1/0	1/8/2	1/1/3
Activity score (A0/A1/A2/A3)	-	2/3/1/5	2/2/1/0

Alanine transaminase, ALT; Aspartate transaminase, AST; Smooth muscle antibody, SMA; Antinuclear antibody, ANA.

## Data Availability

The data presented in this study are available on request from the corresponding author. The data are not publicly available due to restrictions in informed consent.

## Supplementary Materials

The following are available online at https://www.mdpi.com/article/10.3390/cells10102671/s1, Figure S1. Immune checkpoint molecule expression on intra-hepatic NK and NKT cells and the correlation of patient characteristics and intra-hepatic lymphocyte characteristics.
