# Supplementary material for: Increased Intrahepatic Expression of Immune Checkpoint Molecules in Autoimmune Liver Disease
Source: Cells. 2021 Oct 6;10(10):2671. doi: 10.3390/cells10102671 (PMC8534248; doi:10.3390/cells10102671)
Supplement: Supplementary file 1 [file cells-10-02671-s001.zip › cells-1355446-supplementary.pdf]

cells MDPI

Supplementary information

Increased Intrahepatic Expression of Immune Checkpoint Molecules in Autoimmune Liver Disease

Zuzana Macek Jilkova [1,2,3,4,*], Marie Noelle Hilleret [3,4], Theophile Gerster [3,4], Nathalie Sturm [1,5], Marion Mercey-Ressejac [1,2,3], Jean-Pierre Zarski [1,2,3], Vincent Leroy [3], Patrice N. Marche [1,2], Charlotte Costentin [1,2,3,4] and Thomas Decaens [1,2,3,4,*]

1 Université Grenoble Alpes, 38000 Grenoble, France; NSturm@chu-grenoble.fr (N.S.); mressejac@chu-grenoble.fr (M.M.-R.); jpzarski@chu-grenoble.fr (J.-P.Z.); patrice.marche@univ-grenoble-alpes.fr (P.N.M.); CCostentin@chu-grenoble.fr (C.C.)
2 Institute for Advanced Biosciences, Research Center Inserm U1209, CNRS UMR5309, 38700 La Tronche, France
3 Service d'hépato-gastroentérologie, Pôle Digidune, CHU Grenoble Alpes, 38700 La Tronche, France; MNHilleret@chu-grenoble.fr (M.N.H.); tgerster@chu-grenoble.fr (T.G.); vincent.leroy2@aphp.fr (V.L.)
4 Reference Center for Inflammatory Biliary Diseases and Autoimmune Hepatitis (MIVB-H), French Network for Rare Liver Diseases in Children and Adults (FILFOIE), 75012 Paris, France
5 Service d'anatomo-pathologie, Pôle de Biologie, CHU Grenoble Alpes, 38700 La Tronche, France
* Correspondence: zmacekjilkova@chu-grenoble.fr (Z.M.J.); tdecaens@chu-grenoble.fr (T.D.)

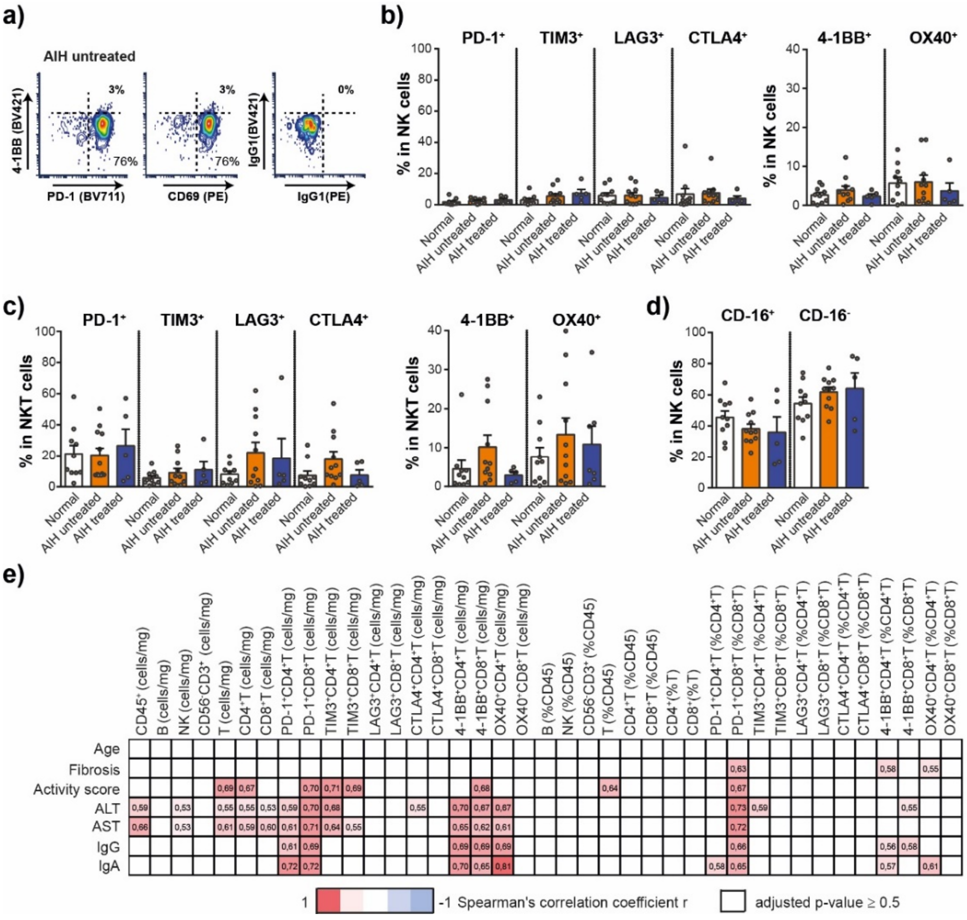

Supplementary Figure S1. BB, PD-1 and CD69 in intrahepatic CD8+ T cells. (**b**) The frequency of intrahepatic immune checkpoint molecule positive NK cells. (**c**) The frequency of intrahepatic immune checkpoint molecule positive NKT (CD56-CD3+ cells). (**d**) The frequency of CD16- and CD16+ NK cells. Normal (n = 10), AIH untreated (n = 11) and AIH treated (n = 5). Each circle represents a patient. Data are expressed as mean ± SEM, (**e**) Correlation of patient characteristics and intrahepatic lymphocyte characteristics in whole cohort, including Normal (n = 10), AIH untreated (n = 11) and

AIH treated (n = 5). Only significant correlations (Bonferroni-corrected p value < 0.5) are reported, numbers correspond to Spearman correlation coefficient r, positive correlation (red), negative correlation (blue).
